# New Appliances and Things Medical

**Published:** 1908-01-25

**Authors:** 


### NEW APPLIANCES AND THINGS MEDICAL.

### THE PULVO.

(The Pulvo Co., 3 Dean Street, High Holborn, W.C.)

We in England, fortunately, have no experience of the typical "dust devil." Unfortunately, however, we have quite enough of its main ingredient, and the religion of asepsis has not gained so many adherents that the laity rigorously condemn the heresy of beating carpets in the street. The ordinary method of dusting a room is one of those illogical and unscientific habits which centuries of authority have ingrained into the average housewife and maid. It would be deemed by these excellent individuals an impertinence on our part to point out to them that the method now in use raises more dust than it removes, but at all events we would cordially recommend them to view The Pulvo.

The Pulvo is a machine that will pick up all the dust in a room and carry it away. It can be used on a hall carpet or on the doctor's best silk hat, and its principle is simplicity itself. It consists essentially of a powerful suction apparatus, operated by means of a flywheel which can be turned by hand or, if desired, by electric power (the plug being fitted to the ordinary household filament lamp-holder), and communicating with a reception-box—the dust-retainer. The air entering the suction-pipe is filtered, and the force of the suction drags up all dust-particles into the collecting-box. The apparatus is simple, efficient, and practical. It accomplishes its work with beautiful effect, and its value, not only to public institutions, but in ordinary households, will be great. The up-to-date armamentarium of the modern housekeeper should include a Pulvo. Prices and catalogue are to be obtained at the London Agency, 3 Dean Street, High Holborn, W.C.

THE HOSPITAL *January* 25, 1908.

